# Comparative Evaluation of Shear Bond Strength of Recycled Metal and Ceramic Orthodontic Brackets Following Different Surface Treatment Methods: An In Vitro Study

**DOI:** 10.7759/cureus.113523

**Published:** 2026-07-28

**Authors:** Leena Gurumayum, Adeel A Bajjad, Manoj K Sharma, Yagya Jain, Anamika Srivastava, Ishika Varshney, Anil Sharma

**Affiliations:** 1 Orthodontics and Dentofacial Orthopedics, Kothiwal Dental College and Research Centre, Moradabad, IND

**Keywords:** ceramic brackets, metal brackets, orthodontic brackets, recycling, sandblasting, shear bond strength

## Abstract

Introduction: Fixed orthodontic treatment relies on adequate bond strength between brackets and enamel to withstand functional forces during therapy. The recycling of debonded brackets is a common practice to reduce treatment costs; however, it may compromise the shear bond strength (SBS) depending on the reconditioning method and bracket material. This in vitro study aimed to evaluate and compare the SBSs of recycled metal and ceramic orthodontic brackets after different surface treatment methods.

Materials and methods: A total of 96 extracted human premolars were randomly divided into two main groups: metal brackets and ceramic brackets (n = 48 each). Each group was further subdivided into four subgroups (n = 12): control (new brackets), sandblasting with 50 µm aluminum oxide, burnout followed by ultrasonic cleaning, and finishing bur. All brackets were bonded using a standard protocol with a light-cured orthodontic adhesive. After debonding, the recycled brackets were treated with the respective surface-conditioning methods and rebonded to fresh teeth. The SBS was tested using a universal testing machine at a crosshead speed of 1 mm/min. Data were analyzed using the Shapiro-Wilk test for normality, two-way analysis of variance (ANOVA), and Tukey’s post-hoc test. Statistical significance was set at p < 0.05.

Results: The new brackets exhibited the highest SBS in both the metal (20.57 ± 1.16 MPa) and ceramic (26.01 ± 1.42 MPa) groups. All recycling methods significantly reduced bond strength compared to the control group. Sandblasting demonstrated superior performance among the recycling techniques, yielding 13.07 ± 2.26 MPa for metal and 20.37 ± 3.41 MPa for ceramic brackets, followed by burnout with ultrasonic cleaning (12.12 ± 0.81 MPa for metal and 14.38 ± 1.64 MPa for ceramic brackets). The finishing bur method resulted in the lowest bond strength values (7.09 ± 1.13 MPa for metal and 14.08 ± 1.49 MPa for ceramics). Two-way ANOVA revealed significant main effects of bracket type and surface treatment, along with a significant interaction between them, indicating that the effect of recycling methods differed between metal and ceramic brackets (p < 0.05).

Conclusion: Recycling orthodontic brackets reduces the SBS, with the magnitude of reduction varying according to the surface treatment method and bracket material. Sandblasting was the most effective in-office recycling technique. Although recycled brackets, especially those treated with sandblasting, may achieve clinically acceptable bond strengths, new brackets are recommended for optimal performance, particularly with ceramic systems. Further in vivo studies are warranted to confirm these findings under clinical conditions.

## Introduction

Orthodontic treatment with fixed appliances relies heavily on the reliable bonding of brackets to the enamel surface to effectively transmit forces from the archwires. Bracket bond failure remains a common clinical challenge, often necessitating replacement or rebonding, which increases treatment time, cost, and patient discomfort [1,2]. Reynolds suggested that a shear bond strength (SBS) of 5.9-7.8 MPa is clinically adequate to withstand masticatory forces, while values exceeding 13 MPa may risk enamel damage [3].

Metal brackets have traditionally been widely used because of their durability, predictable bond strength, and cost-effectiveness. However, the growing demand for esthetics has popularized ceramic brackets, which offer a superior appearance but present challenges such as brittleness and higher initial bond strengths that can complicate debonding [4]. Bond failures or placement errors frequently require orthodontists to recycle previously debonded brackets [5]. Recycling methods aim to remove residual adhesive while preserving the bracket’s integrity and retentive properties. Common in-office techniques include mechanical removal with a finishing bur, sandblasting with aluminum oxide particles, and thermal burnout, followed by ultrasonic cleaning [6,7].

Recycling offers economic benefits by reducing the need for new brackets; however, it can alter the bracket base morphology, surface roughness, and ultimately the SBS [6]. The literature shows variable outcomes depending on the bracket material and recycling protocol. Some studies have reported clinically acceptable SBS after sandblasting, whereas others have noted significant reductions with thermal or mechanical methods, particularly for ceramic brackets [6-9].

This in vitro study aimed to evaluate and compare the SBSs of metal and ceramic orthodontic brackets recycled using three different surface treatment methods-finishing bur, sandblasting with 50 µm aluminum oxide, and burnout followed by ultrasonic cleaning-against new (control) brackets bonded with Transbond XT composite. Specifically, this study assessed the bond strength achieved with new brackets and determined the relative effectiveness of each recycling technique in restoring clinically acceptable SBS for both bracket types.

## Materials and methods

This in vitro experimental study was conducted in the Department of Orthodontics and Dentofacial Orthopedics at Kothiwal Dental College and Research Centre, Moradabad, Uttar Pradesh, India, between March 2024 and December 2024. The study protocol was reviewed and approved by the Institutional Ethics Review Board of Kothiwal Dental College and Research Centre (reference no. KDCRC/IERB/02/2024/18 dated February 20, 2024). 96 extracted human premolars were collected for the study. Teeth and brackets were selected based on the eligibility criteria (Table 1).

**Table 1:** Inclusion and exclusion criteria for tooth and bracket selection

Inclusion criteria	Exclusion criteria
Premolar teeth with intact buccal enamel surfaces	Developmental defects in enamel
Extraction for orthodontic or periodontal purposes	Carious tooth
Vitality at the time of extraction	Restored tooth
New metal brackets without any breakage or distortion	Previously bonded brackets
New ceramic brackets without any breakage or distortion	Previous chemically treated brackets

A priori sample size calculation was performed using G*Power software (version 3.1, Heinrich-Heine-Universität Düsseldorf, Germany). Based on parameters of bond strength for recycled ceramic brackets, derived from Han et al. [10], an expected effect size (Cohen’s d) of 0.55, a minimum of 42 specimens per group (total 84 specimen) was required for a two-tailed independent t-test with α = 0.05 and a power of 80%. Accounting for a 10% anticipated attrition rate and subgroup analysis, the sample size was increased to 48 specimens per group, resulting in a total of 96 samples (48 metal brackets and 48 ceramic brackets).

**Figure 1:**
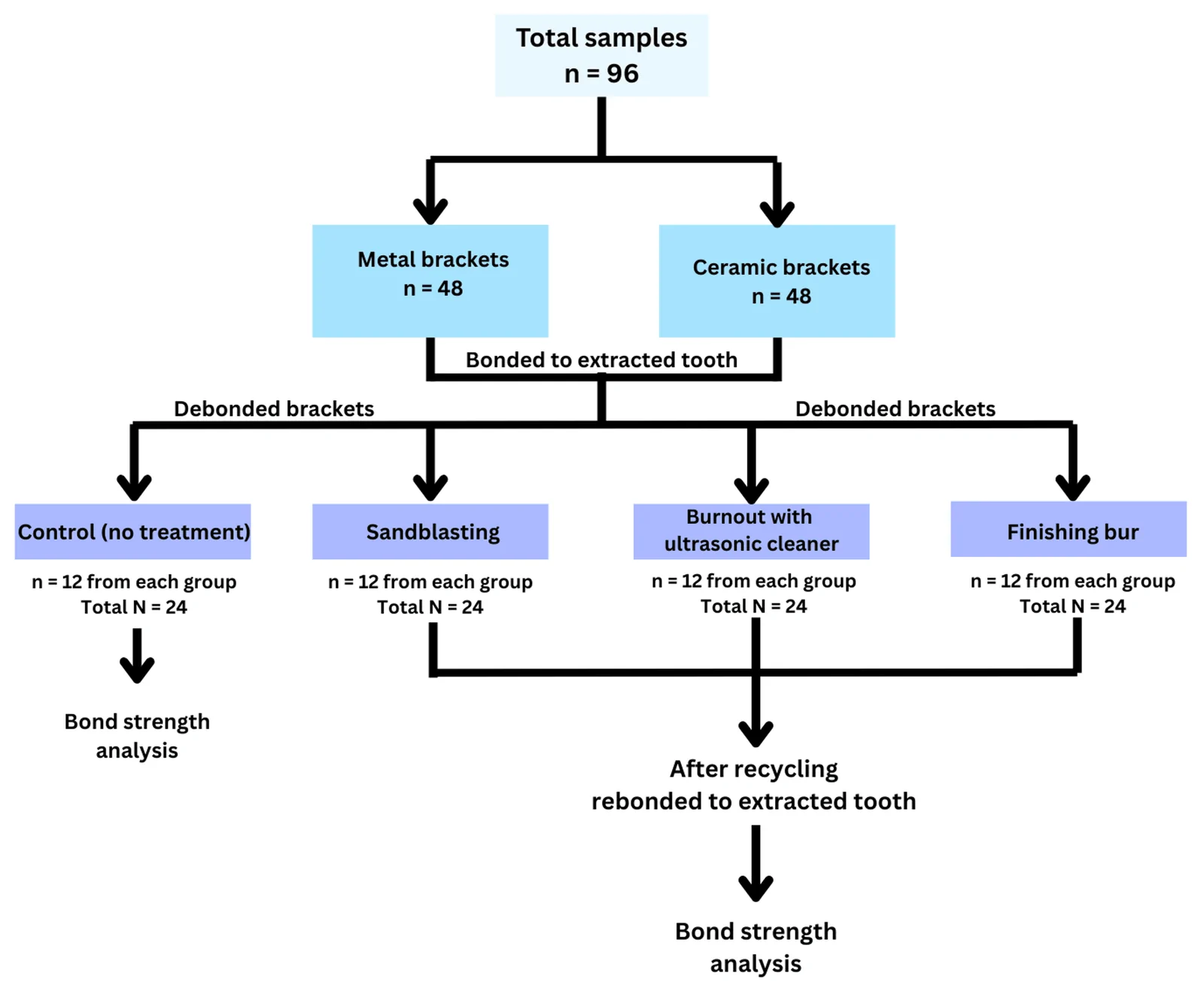
Study flow chart and group assignment Image created with Canva.com (Canva, Australia)

The selected premolars were randomly divided into two main groups of 48 teeth each: Group I for metal brackets (0.022” MBT prescription, Libral, India) and Group II for ceramic brackets (0.022” MBT prescription, Libral, India). Each main group was further subdivided into four subgroups (n = 12 per subgroup). Subgroup A served as the control using new brackets, while Subgroups B, C, and D underwent recycling by sandblasting, burnout with ultrasonic cleaning, and finishing bur, respectively (Figure 1). All specimens were bonded using Transbond XT light-cured orthodontic adhesive and primer (3M Unitek, USA).

The bonding procedure was standardized across all groups. The buccal surfaces of the teeth were etched with 37% phosphoric acid (3M Unitek, USA) for 15 s, rinsed with water for 15 s, and air-dried until a frosty white appearance was obtained. A thin layer of Transbond XT primer was applied to the etched enamel, followed by the placement of the Transbond XT adhesive on the bracket base. The bracket was firmly seated on the tooth surface, and excess adhesive was removed using an explorer. Light curing was performed for 40 s (10 s each from the mesial, distal, occlusal, and gingival directions) using an LED curing light. The bonded specimens were stored in distilled water at 37°C for 24 h.

After initial debonding, the brackets in the recycling subgroups were treated as follows. For sandblasting, residual adhesive was removed using 50 µm aluminum oxide particles (Aluminum Oxide Abrasive, Danville Materials, USA) delivered through a micro-sandblaster (MicroEtcher II, Danville Materials, USA) at a distance of 10 mm until the bracket base was clean, followed by rinsing with water and air drying. For the burnout method, brackets were flame-treated in the reducing zone of a gas microtorch (Orca Gas Micro Torch, Blazer Products Inc., USA) for 5 seconds, quenched in room-temperature water, and then ultrasonically cleaned for 10 minutes in an ultrasonic cleaner (Elmasonic S, Elma Schmidbauer GmbH, Germany) containing a dedicated cleaning solution. For the finishing bur group, the residual composite was carefully removed using an extra-fine grit diamond finishing bur (Mani Inc., Japan) under continuous water cooling in unidirectional strokes until no visible adhesive remained. All recycled brackets were rebonded to new premolars using the identical standardized bonding protocol and stored in distilled water for another 24 hours. SBS testing was performed using a universal testing machine (Instron 3366, Instron Corporation, USA) at a crosshead speed of 1 mm/min. The debonding force was recorded in N and converted to MPa by dividing it by the bracket base area.

Statistical analyses were performed (IBM SPSS Statistics for Windows, Version 25.0, IBM Corp., USA). The normality of the data distribution was assessed using the Shapiro-Wilk test. Descriptive statistics were expressed as mean ± SD, along with 95% confidence intervals. A two-way analysis of variance (ANOVA) was used to evaluate the main effects of the bracket type, surface treatment, and their interactions. When significant differences were detected, Tukey’s post-hoc multiple comparison test was applied after adjusting for multiple comparisons. A p < 0.05 was considered statistically significant.

## Results

The normality of the data distribution was assessed using the Shapiro-Wilk test. Most variables, including the SBS, followed a normal distribution, allowing the use of parametric tests. The descriptive statistics for the SBS of metal and ceramic brackets treated with different surface treatment methods are presented in Table 2. For metal brackets, the control group showed the highest mean SBS (20.57 ± 1.16 MPa), followed by sandblasting (13.07 ± 2.26 MPa), burnout with ultrasonic cleaning (12.12 ± 0.81 MPa), and the bur group, which recorded the lowest value (7.09 ± 1.13 MPa). For ceramic brackets, the control group demonstrated the highest mean SBS (26.01 ± 1.42 MPa), followed by sandblasting (20.37 ± 3.41 MPa), burnout with ultrasonic cleaning (14.38 ± 1.64 MPa), and the bur group (14.08 ± 1.49 MPa).

**Table 2:** Descriptive statistics for SBS (MPa) of metal and ceramic brackets treated with different methods Data are presented as mean ± SD Group: Type of bracket, Subgroup: Type of surface treatment for recycling SBS: Shear bond strength

Group, (n = 48 each)	Subgroup, (n = 12 each)	95% confidence interval for mean	Mean ± SD
Metal bracket	Control (no treatment)	19.83-21.3	20.57 ± 1.16
	Sandblasting	11.63-14.51	13.07 ± 2.26
	Burnout and ultrasonic cleaner	11.6-12.63	12.12 ± 0.81
	Finishing bur	6.38-7.81	7.09 ± 1.13
Ceramic bracket	Control (no treatment)	25.1-26.91	26.01 ± 1.42
	Sandblasting	18.21-22.54	20.37 ± 3.41
	Burnout and ultrasonic cleaner	13.34-15.42	14.38 ± 1.64
	Finishing bur	13.14-15.03	14.08 ± 1.49

Two-way ANOVA (Table 3) revealed statistically significant main effects for the type of bracket (F = 215.39, p < 0.001, ηp² = 0.71), treatment (F = 214.95, p < 0.001, ηp² = 0.88), and their interaction (F = 9.48, p < 0.001, ηp² = 0.24). These results indicate that both the bracket material and surface treatment significantly influenced the SBS, with the effect of treatment varying between metal and ceramic brackets.

**Table 3:** Results of two-way ANOVA for the effect of bracket type and surface treatment on SBS *Statistically significant at p < 0.05, ηp² = partial eta squared. ANOVA: Analysis of variance; SBS: Shear bond strength

Source of variation	Type III sum of squares	df	Mean square	F value	p-value	ηp²
Type of bracket	725.34	1	725.34	215.39	< 0.001*	0.71
Treatment	2171.53	3	723.84	214.95	< 0.001*	0.88
Type of bracket x Treatment	95.75	3	31.92	9.48	< 0.001*	0.24
Error	296.34	88	3.37	-	-	-

Post-hoc analysis with Tukey-adjusted pairwise comparisons (Table 4) showed that the control groups of both metal and ceramic brackets had significantly higher SBS than all recycled groups (p < 0.001). Among the recycled metal brackets, sandblasting and burnout with ultrasonic cleaning produced significantly higher bond strengths than the bur method (p < 0.001), with no significant difference between the sandblasting and burnout groups. For ceramic brackets, sandblasting resulted in significantly higher SBS than both the burnout with ultrasonic cleaning and bur methods (p < 0.001). The control ceramic brackets exhibited the highest overall bond strength, whereas the bur-treated metal brackets exhibited the lowest values. Several pairwise comparisons between the recycled groups and the ceramic control were also statistically significant. In summary, all recycling methods led to a significant reduction in the SBS compared to new brackets. Sandblasting was the most effective recycling method among the tested techniques, particularly for ceramic brackets, whereas the finishing bur method resulted in the greatest reduction in SBS for both bracket types.

**Table 4:** Tukey post-hoc pairwise comparisons of SBS between different groups *Statistically significant at p < 0.05 Only statistically significant comparisons (p < 0.05) are highlighted in the full analysis. SBS: Shear bond strength

Pairwise comparison		Mean difference (MPa)	t statistics	p-value
Control - Metal	Bur - Metal	13.47	17.99	< 0.001*
Control - Metal	Sandblast - Metal	7.49	10.00	< 0.001*
Control - Metal	Burnout - Metal	8.45	11.28	< 0.001*
Control - Metal	Control - Ceramic	-5.44	-7.26	< 0.001*
Control - Metal	Bur - Ceramic	6.48	8.66	< 0.001*
Control - Metal	Sandblast - Ceramic	0.19	0.26	1.000
Control - Metal	Burnout - Ceramic	6.19	8.26	< 0.001*
Bur - Metal	Sandblast - Metal	-5.98	-7.98	< 0.001*
Bur - Metal	Burnout - Metal	-5.03	-6.71	< 0.001*
Bur - Metal	Control - Ceramic	-18.92	-25.25	< 0.001*
Bur - Metal	Bur - Ceramic	-6.99	-9.33	< 0.001*
Bur - Metal	Sandblast - Ceramic	-13.28	-17.73	< 0.001*
Bur - Metal	Burnout - Ceramic	-7.29	-9.73	< 0.001*
Sandblast - Metal	Burnout - Metal	0.95	1.27	1.000
Sandblast - Metal	Control - Ceramic	-12.93	-17.26	< 0.001*
Sandblast - Metal	Bur - Ceramic	-1.01	-1.35	1.000
Sandblast - Metal	Sandblast - Ceramic	-7.30	-9.74	< 0.001*
Sandblast - Metal	Burnout - Ceramic	-1.31	-1.74	1.000
Burnout - Metal	Control - Ceramic	-13.89	-18.54	< 0.001*
Burnout - Metal	Bur - Ceramic	-1.96	-2.62	0.290
Burnout - Metal	Sandblast - Ceramic	-8.25	-11.02	< 0.001*
Burnout - Metal	Burnout - Ceramic	-2.26	-3.02	0.094
Control - Ceramic	Bur - Ceramic	11.92	15.92	< 0.001*
Control - Ceramic	Sandblast - Ceramic	5.63	7.52	< 0.001*
Control - Ceramic	Burnout - Ceramic	11.63	15.52	< 0.001*
Bur - Ceramic	Sandblast - Ceramic	-6.29	-8.40	< 0.001*
Bur - Ceramic	Burnout - Ceramic	-0.30	-0.40	1.000
Sandblast - Ceramic	Burnout - Ceramic	5.99	8.00	< 0.001*

## Discussion

This in vitro study evaluated the effects of three different surface treatment methods on the SBS of recycled metal and ceramic orthodontic brackets. Consistent with the existing literature, new brackets demonstrated superior bond strength compared to recycled ones, highlighting the inevitable impact of recycling procedures on adhesive performance [11]. Ceramic brackets generally exhibit higher bond strength than metal brackets in the new condition, which can be attributed to their chemical retention mechanisms and greater surface area available for bonding [12]. However, recycling produced a more noticeable decline in the performance of ceramic brackets, likely because of their inherent brittleness and susceptibility to surface alterations [13].

Among the recycling techniques, sandblasting was the most effective method for restoring bond strength in both bracket types. A recent systematic review and meta-analysis by Anita and Kailasam concluded that sandblasting is an efficient method for recycling debonded metal brackets, with no clinically significant difference in SBS compared with new brackets [14]. This supports the findings of the present study, reinforcing sandblasting as a reliable, in-office recycling technique. Similarly, Quick et al. concluded that sandblasting was superior to flaming or mechanical grinding for stainless steel brackets because it cleans the base thoroughly while preserving the mesh structure [15]. The abrasive nature of sandblasting removes residual adhesive without causing excessive damage to the retentive features, allowing better adhesive penetration. Toroglu and Yaylali reported that sandblasting combined with silane application on debonded mechanically retentive ceramic brackets produced bond strengths comparable to new brackets [16]. Their study further demonstrated that silica coating followed by silane application yielded even higher bond strength values, supporting the effectiveness of surface conditioning methods in restoring the retentive capacity of the recycled ceramic brackets.

The inferior performance of the finishing bur method observed in the present study is consistent with the findings of Basudan and Al-Emran [4]. In their evaluation of five in-office reconditioning techniques, adhesive grinding using a green stone resulted in a significant 28% reduction in shear/peel bond strength compared with new brackets. The authors attributed this decline to the smoothing effect on the bracket base, which reduces the surface irregularities and micromechanical retention necessary for optimal adhesive bonding. Scanning electron microscopic analysis in their study further revealed that, while the wire mesh structure was generally preserved, the amount of residual adhesive and surface characteristics varied considerably depending on the reconditioning method. Mechanical grinding with stones or burs, similar to the finishing bur technique used in the current study, was less effective than sandblasting or other methods. These observations support the conclusion that aggressive mechanical removal of adhesive with burs should be avoided when recycling brackets if an adequate bond strength is desired [17].

Burnout followed by ultrasonic cleaning produced intermediate results. Thermal recycling can effectively eliminate residual composites; however, the heat may induce microstructural changes in metal brackets or cause microcracks in ceramic materials. Subsequent ultrasonic cleaning helps remove debris; however, the overall bond strength recovery remains lower than that of sandblasting. These observations are supported by Chacko et al. and Mirhashemi et al., who noted moderate outcomes with combined thermal and ultrasonic protocols [17,18]. A recent systematic review and meta-analysis by Grazioli et al. reported that direct flame treatment of debonded metal brackets resulted in significantly lower bond strength than that of new brackets [19]. This supports the moderate performance of the burnout method observed in the present study, indicating that while thermal recycling is clinically acceptable, it is less effective than sandblasting in restoring optimal SBS.

The significant interaction between bracket type and treatment method observed in the present study indicates that ceramic brackets respond differently to recycling procedures than metal brackets. This differential effect has been documented in previous studies, which attributed it to the brittle nature of ceramics versus the more forgiving ductile properties of stainless steel [20,21]. The findings of this study are generally in agreement with the broader literature, which shows that while recycling offers economic advantages, it consistently leads to a reduction in bond strength. However, sandblasting appears to be the most reliable in-office method for maintaining clinically acceptable bond strength values. Differences across studies may arise from variations in bracket designs, adhesive systems, particle sizes used for sandblasting, duration of thermal application, and storage conditions.

Clinical implications

Recycled orthodontic brackets treated with sandblasting can be considered for clinical use, particularly in situations where cost-effectiveness is important or for short-term applications. However, new brackets are recommended when maximum bond reliability is required, especially with esthetic ceramic brackets. Clinicians should prefer sandblasting over bur or thermal methods when recycling is necessary and ensure proper case selection to minimize the risk of bond failure.

Limitations

Being an in vitro study, the present investigation has certain limitations. It could not simulate the full complexity of the oral environment, including long-term thermocycling, saliva contamination, masticatory forces, and microbial interactions. The study evaluated only single-cycle recycling, whereas brackets may be recycled multiple times in clinical settings. In addition, adhesive remnant index (ARI) scoring, bracket surface morphology assessment, and scanning electron microscopy (SEM) evaluation were not performed. Consequently, the mechanisms underlying the observed differences in SBS, including the effects of surface alterations and residual adhesive on bracket retention, could not be fully elucidated. Future research incorporating aging protocols, multiple recycling cycles, ARI analysis, SEM-based surface characterization, and well-designed in vivo clinical trials would provide more comprehensive insights into the clinical performance of recycled brackets.

## Conclusions

Within the limitations of this in vitro study, all recycling methods significantly reduced the SBS of both metal and ceramic orthodontic brackets compared with new brackets. Among the evaluated surface treatment methods, sandblasting demonstrated the highest bond strength, followed by burnout with ultrasonic cleaning, whereas the finishing bur method produced the least favorable results. Recycled brackets treated with sandblasting may be considered clinically acceptable because they achieved bond strengths within the clinically acceptable range; however, these findings should be interpreted with caution and require confirmation through well-designed in vivo clinical studies before routine clinical adoption. New brackets remain the preferred option when maximum bond reliability is required, particularly for ceramic bracket systems.

## References

[1] Quinty O, Antonarakis GS, Kiliaridis S, Mavropoulos A (2024). Factors related to bracket bond failure during orthodontic treatment: A single-centre single-operator study. Dent J (Basel).

[2] Sukhia RH, Sukhia HR, Azam SI, Nuruddin R, Rizwan A, Jalal S (2019). Predicting the bracket bond failure rate in orthodontic patients: a retrospective cohort study. Int Orthod.

[3] Reynolds IR, von Fraunhofer JA (1976). Direct bonding of orthodontic brackets - a comparative study of adhesives. Br J Orthod.

[4] Basudan AM, Al-Emran SE (2001). The effects of in-office reconditioning on the morphology of slots and bases of stainless steel brackets and on the shear/peel bond strength. J Orthod.

[5] Jakavičė R, Kubiliūtė K, Smailienė D (2023). Bracket bond failures: incidence and association with different risk factors - a retrospective study. Int J Environ Res Public Health.

[6] Khanal PP, Shrestha BK, Yadav R, Prasad Gupta S (2021). A comparative study on the effect of different methods of recycling orthodontic brackets on shear bond strength. Int J Dent.

[7] Kumar M, Maheshwari A, Lall R, Navit P, Singh R, Navit S (2014). Comparative evaluation of shear bond strength of recycled brackets using different methods: an in vitro study. J Int Oral Health.

[8] Nawrocka A, Nowak J, Sauro S, Hardan L, Bourgi R, Lukomska-Szymanska M (2023). Shear bond strength of metal and ceramic brackets depending on etching protocol in direct bonding technique. Materials (Basel).

[9] Zheng X, Huang X (2024). Evaluation of the re-bond strength of debonded metal and ceramic brackets following Er: YAG laser treatment. BMC Oral Health.

[10] Han RQ, Yang K, Ji LF, Ling C (2016). Analysis of shear bond strength and morphology of Er:YAG laser-recycled ceramic orthodontic brackets. Biomed Res Int.

[11] Gupta N, Kumar D, Palla A (2017). Evaluation of the effect of three innovative recyling methods on the shear bond strength of stainless steel brackets-an in vitro study. J Clin Exp Dent.

[12] Forsberg CM, Hagberg C (1992). Shear bond strength of ceramic brackets with chemical or mechanical retention. Br J Orthod.

[13] Martina R, Laino A, Cacciafesta V, Cantiello P (1997). Recycling effects on ceramic brackets: a dimensional, weight and shear bond strength analysis. Eur J Orthod.

[14] Anita P, Kailasam V (2021). Effect of sandblasting on the shear bond strength of recycled metal brackets: a systematic review and meta-analysis of in-vitro studies. Int Orthod.

[15] Quick AN, Harris AM, Joseph VP (2005). Office reconditioning of stainless steel orthodontic attachments. Eur J Orthod.

[16] Toroglu MS, Yaylali S (2008). Effects of sandblasting and silica coating on the bond strength of rebonded mechanically retentive ceramic brackets. Am J Orthod Dentofacial Orthop.

[17] Chacko PK, Kodoth J, John J, Kumar K (2013). Recycling stainless steel orthodontic brackets with Er:YAG laser - an environmental scanning electron microscope and shear bond strength study. J Orthod Sci.

[18] Mirhashemi AH, Hosseini MH, Chiniforoush N, Soudi A, Moradi M (2018). Shear bond strength of rebonded ceramic brackets using four different methods of adhesive removal. J Dent (Tehran).

[19] Grazioli G, Hardan L, Bourgi R (2021). Residual adhesive removal methods for rebonding of debonded orthodontic metal brackets: systematic review and meta-analysis. Materials (Basel).

[20] Lew KK, Chew CL, Lee KW (1991). A comparison of shear bond strengths between new and recycled ceramic brackets. Eur J Orthod.

[21] Karamouzos A, Athanasiou AE, Papadopoulos MA (1997). Clinical characteristics and properties of ceramic brackets: a comprehensive review. Am J Orthod Dentofacial Orthop.

